# Exploring the views of stakeholders about the feasibility of carrying out a randomised controlled trial of Individual Placement and Support for people unemployed with chronic pain based in primary care (the InSTEP study)

**DOI:** 10.1186/s40814-020-00588-z

**Published:** 2020-04-04

**Authors:** Michelle M. Holmes, Sabina C. Stanescu, Catherine Linaker, Catherine Price, Nick Maguire, Simon Fraser, Cyrus Cooper, Karen Walker-Bone

**Affiliations:** 1 Psychology, University of Southampton, Southampton, SO17 1BJ UK; 2 Arthritis Research UK/MRC Centre for Musculoskeletal Health and Work, Southampton General Hospital, University of Southampton, Southampton, SO16 6YD UK; 3 Medical Research Council Lifecourse Epidemiology Unit, Southampton General Hospital, University of Southampton, Tremona Road, Southampton, SO16 6YD UK; 4 Academic Unit of Primary Care and Population Sciences, Faculty of Medicine, Southampton General Hospital, University of Southampton, Southampton, SO16 6YD UK; 5 Solent NHS Trust, Highpoint Venue, Bursledon Rd, Southampton, SO19 8BR UK

**Keywords:** Unemployment, Chronic pain, Individual Placement and Support (IPS), Vocational rehabilitation, Feasibility

## Abstract

**Background:**

Individual Placement and Support (IPS) is a model of vocational rehabilitation originally developed to help people with severe mental illness obtain and maintain employment. Work disability is common amongst people with chronic pain conditions, yet few effective interventions exist. As part of mixed-methods feasibility research and as a forerunner to a pilot trial (In STEP), we investigated the barriers and facilitators to carrying out a future randomised controlled trial of IPS set in primary care amongst people unemployed with chronic pain.

**Methods:**

Semi-structured interviews and focus groups were conducted with: unemployed people with health conditions receiving IPS (clients), Employment Support Workers (ESWs) delivering IPS for people with chronic health conditions and primary healthcare professionals. Interviews and focus groups were transcribed verbatim and analysed with field notes using thematic analysis.

**Results:**

All stakeholders generally viewed a future trial of IPS positively and deemed both the intervention and treatment as usual acceptable. Themes that emerged regarding potential barriers were recruitment, the importance of recruiting people voluntarily who wanted to return to work and were motivated to do so and giving them agency in the process; a need for additional training and support of the ESWs; and a risk of over-burdening participants with paperwork. Regarding facilitators however, the themes were offering the intervention early after unemployment, the importance of relationship and continuity with the ESWs and that an employment intervention could bring a range of health benefits.

**Conclusions:**

All stakeholders thought that a randomised trial was potentially feasible and highlighted some potential advantages of participation.

**Trial registration:**

Study no ISRCTN30094062

## Background

Chronic pain is pain persistent beyond the usual period of healing, usually defined as 3 months [1]. It is common, affecting an estimated 35.0–51.3% of the UK population [2] at any point in time. The prevalence increases with age [2] and is more common and more severe amongst those of lower socioeconomic status [3]. Although people may recover from chronic pain, the prognosis is poor after several years, with psychosocial factors implicated strongly [4]. Therefore, chronic pain is a major health problem associated with impaired function and poor quality of life [5]. Healthcare costs are also high: chronic pain patients consult in primary care up to five times more frequently than others, equating to almost 5 million appointments annually in the UK [6].

The functional impact of chronic pain is thought to be even more costly: between 20–27% of people of working age with chronic pain are unable to participate in their usual activities, including work, because of their pain. An estimated 48–88% of the total cost burden of chronic pain is indirect costs arising from restricted productivity, sick leave, disability benefits and other aspects of work disability. Prolonged unemployment, for any reason, causes additional health problems. Those who lose their job suffer from worse mental health [7], poorer life expectancy [8], attend healthcare consultations more frequently and report higher levels of pain [9]. Moreover, the children of unemployed people also have poorer mental health and themselves experience higher rates of unemployment [10]. Taken together, these findings illustrate the potential public health impact of rehabilitating people with chronic pain back into work.

An approach that has been successful in enabling occupational rehabilitation for people with severe mental health conditions is Individualised Placement Support (IPS) [11–13]. IPS is based around a “place and train” model, which focuses on finding rapid employment in the competitive labour market for clients based on their clinical needs and preferences, and providing ongoing support from an employment support worker (ESW) that takes into account and integrates with the medical needs of the client.

Despite the success of this approach amongst people with severe mental ill-health [11–13], this approach has not yet been tested much for other health conditions. One recent Norwegian pilot study evaluated IPS amongst 8 chronic pain patients and found positive impacts, particularly on function [14]. There was one drop out, but 3/8 obtained competitive employment over 12 months, and a full randomised controlled trial (RCT) [15] is currently in progress. However, the health, social and welfare systems in Norway and the UK are very different and what works in Norway may not necessarily transfer to a very differently organised health and social care system. Therefore, complementary research is required to demonstrate whether this approach can be effective at returning people to the workplace and cost effective to the healthcare system.

In planning trials to assess complex interventions such as IPS, it is helpful to establish their practicability through a preliminary study, which can help to: refine the intervention, determine the feasibility of recruitment, inform inclusion/exclusion criteria, establish the acceptability of randomization and provide estimates of retention rates and the prevalence of suitable outcome measures to inform future power calculations [16–18]. As part of the feasibility research, stakeholder views on the methodology and procedures proposed for a full-scale trial can be invaluable. To our knowledge, no published studies have explored the views of multiple stakeholders regarding IPS for chronic pain. Therefore, as a forerunner to a pilot study, we undertook a qualitative study to explore the barriers to, and facilitators of, engaging individuals with chronic pain in a future trial of IPS. Specifically, our aims were to explore stakeholders’ overall views about a trial using IPS; evaluate possible methods of recruitment; identify any barriers to, and facilitators of, participation in a future trial for recruiters and patients; understand what outcomes of such an intervention are of greatest importance to patients; understand views from stakeholders about how the existing local IPS programme might need adaptation for a trial in chronic pain; and establish what, if any, additional needs there would be for ESWs supporting clients through IPS.

## Methods

### Participants

The study recruited samples from three key stakeholder groups (clients, Employment Support Workers (ESWs) and healthcare professionals (HCPs)).

The opportunity for this feasibility research was created by the availability of a local IPS programme (the Solent Jobs Programme (SJP)). This programme was funded by the European Social Fund and matched City Deal/local funds to offer IPS to people with any chronic health condition (including pain) who had been unemployed ≥ 2 years. To enter the SJP, clients were mostly identified through job centres and engagement was voluntary. For the current research, we asked ESWs and SJP managers to identify clients who were currently engaging with IPS and were willing to speak about their experiences to independent researchers who have nothing to do with programme delivery. We provided a patient information leaflet (PIL) which explained the nature and purpose of our research. We did not purposively sample for any age, gender or health condition so as to collect a range of views from people actually engaging with IPS about what would enable or prevent them from participating in a research project which offered IPS as the active arm of the intervention. ESWs and programme managers were asked to hand out the PIL and collect the contact details of any IPS client who gave their consent for contact information to be passed to the research team who would then contact them about taking part in an interview. Willing participants were contacted by telephone by the researcher to arrange the interview. Interviews were carried out at a time convenient to the participants, and they were offered a choice of an interview by telephone or face-to-face in their local job centre. It was explained to clients that the interviewers were independent from the IPS programme, and that they were part of a research team investigating whether high-quality employment services could help the health of unemployed people with long-term conditions. All were assured that their comments would be confidential. Nobody from the IPS programme was present during data collection. Travel expenses were remunerated, and each client was sent a food voucher after their interview to thank them for their participation.

All ESWs involved in IPS were eligible, but the pool of ESWs currently involved with the programmes was relatively small. Participation in the research was discussed with each ESW and it was explained that their participation was entirely voluntary and that their comments would be anonymised and non-attributable. Interviews were arranged by the researcher at their convenience and at their place of work. Travel expenses were remunerated.

Primary healthcare providers were contacted through the local Clinical Research Network who advertised the study to research-active practices. Practices that expressed interest were then contacted by a member of the research team by telephone to arrange a suitable time for a focus group. Participating general practices were remunerated so that they could backfill the time required. For convenience, the views of healthcare providers were elicited at focus groups, reducing the total clinical time used. Focus groups took place at a convenient time within the practice premises. Members of the primary healthcare team involved in the care of chronic pain patients were eligible (including doctors, nurses, physiotherapists and practice managers).

All potential participants were provided with written information about the study in advance. The information explained the purpose of the study, what was required of participants, that their expressed views would be confidential, anonymised and non-attributable, and that they could withdraw their consent at any time. Written, informed consent to participate, and for the analysis of their views, was obtained from all participants.

This research received ethical approval from the University of Southampton Research Ethics Committee (ID 23853) and research governance approval from the Health Regulatory Authority (17/HRA/0035) in January 2017.

### Data collection

The research took a qualitative approach, using semi-structured interviews, focus groups and qualitative field notes. Interviews allow for participants to tell the story of their own experiences [19, 20]. Focus groups were used to facilitate interaction between participants, enabling them to bounce ideas off each other [21]. Two female PhD students with prior experience in qualitative data collection (MH, SS) conducted the interviews and focus groups, following a semi-structured topic guide (see Table 1). Broadly, the aim of all interviews was to capture the participants’ views about barriers to and facilitators of a future RCT in which IPS was the active intervention. Neither interviewer had any prior relationship with the participants. Questions and prompts were developed in advance to aid the interviewer, but the topic guide was intentionally flexible to allow for natural discussion throughout the process. Field notes were made by the interviewers during and immediately after data collection.

**Table 1:** Summary of interview topics

Clients	Employment Support Workers	Healthcare professionals
• Experience of IPS (e.g. Can you tell me about the programme?) • Research on IPS (e.g. What would be important when planning a trial for similar support programme ?) • Recruitment (e.g. How did you get to be involved in the programme?) • Outcomes of IPS (e.g. What do you think you have got out of the programme?)	• Experience of IPS (e.g. Can you tell me about your role in the programme?) • Research on IPS (e.g. What would be important when planning a trial for similar support programme ?) • Recruitment (e.g. What do you think makes people decide to take part?) • Integration with pain services (e.g. What support do you think you need to work with existing services?) • Outcome of IPS (e.g. What do you think clients get out of the programme?) • Barriers (e.g. Can you identify any issues when planning a new trial for patients with chronic pain?)	• Recruitment (e.g. What are your thoughts on identifying and recruiting patients with chronic pain who are unemployed?) • Outcomes of IPS (e.g. Do you think this programme might be beneficial to patients?) • Integration of IPS with pain services (e.g. How could IPS work with primary care and existing services?) • Control intervention (e.g. What do you think about this being an RCT with a control group?)

### Data analysis

Interviews and focus groups were transcribed verbatim by two interviewers, and all text was entered into the NVivo qualitative data analysis software version 11 for analysis [22]. The data were analysed thematically independently [23]. Each interview and focus group was coded by both researchers. Refinement and grouping of initial codes into higher-level categories was conducted by discussion between both researchers: (1) the data were coded inductively, (2) codes were examined for patterns and refined, (3) relationships and refined patterns between codes were identified and themes were developed, and (4) themes were described with representative data to support the theme. This methodology enabled thorough exploration and detailed description of stakeholders’ views [24, 25]. Quotes have been selected from the arising themes to best describe the findings, with non-identifiable ID numbers allocated to participants. The study findings are reported according to the consolidated criteria for reporting qualitative studies (COREQ) [26].

## Results

Data collection took place in 2017. During 2017, approximately 50 people per month were referred to Solent Jobs Programme, just under half of whom agreed to join the programme. Overall, more men than women participated, two-thirds were aged 18–49, and most (> 90%) were white British. The most common level of qualification (41%) was school level. During the recruitment for our research, 20 potential IPS clients registered their interest to participate. However, 5 proved to be uncontactable by telephone to arrange an interview, and 6 failed to attend at the time agreed. Therefore, in total, nine client interviews were carried out (five males, four females, all unemployed more than 2 years). Interviews lasted a maximum of 45 min.

Two GP practices, including 11 healthcare professionals (HCPs), took part in two focus groups. The composition of these groups (doctors, nurses, physiotherapists, practice managers, etc.) was based on availability, which was timed to make the groups as inclusive and accessible as possible. Focus groups were set up to last for a maximum of 1 h.

Eight ESWs at two IPS sites were given information about the research. Six ESWs expressed interest in participating. However, due to their work schedule, one was unable to take part. Thus, five interviews were carried out, none of which took longer than 45 min.

### Individual Placement and Support

In general, all stakeholders were very positive about IPS, with clients and ESWs sharing success stories and the benefits of the programme. This positive view was also shared by the HCPs: “I think the intentions are brilliant.” (HCP1).

However, clients found it difficult to separate out talking about a potential research trial from their personal experiences of their current IPS programme. Even after clarification, participants seemed to find the concept of a trial difficult to understand and their answers regarding research on IPS were inherently linked to their experience of IPS. An example of this is when discussing recruitment to a trial. Clients seemed anxious that recruitment to a trial would mean additional workloads for their ESWs, who they perceived already had limited time available without adding new clients in a research trial. “I mean at the end of the day they’ve got 25-30, erm, clients, and you’ve only got four advisors maybe five advisors. And to be totally honest, they are also doing more interviews at the job centre in [location] and [location] and that to get more people on this course. So you are limited to the amount of time you can have with them.” (C2).

One client acknowledged the benefit of conducting the proposed trial but expressed concerns about the purpose of the research. She expressed concerns about the wording of the information sheet which stated the research aim was to “improve the health of people who are unemployed through long-term health problems”. She felt this was derogatory, feeling that she was not “unemployed” but “unable to work”. At the end of the interview, she voiced apprehensions about the future study findings forcing individuals to work even if they were unable: “I think this is a really interesting study. I think that, it’s important, but I also think in the wrong hands, it could be used to be malicious and force people with pain into work.” (C5).

Additionally, HCPs stated that they had seen patients who were trying to get back to work and find employment, and that these would be interested and engaged. However, they discussed their worries that some individuals might not wish to return to work and that differences in motivation would be a problem for the trial. “And it’s the individuals, there are some people who actually like to work as we would like to see that, but there are those who just wanna get the money and the money is just easier if you are just sitting on your backside sometimes.” (HCP1).

In summary, in principle, research on IPS for individuals with chronic pain was seen positively. However, framing research in this field to clients proved problematic, and clients struggled to understand the purpose of their interviews in the future research. HCPs also highlighted potential difficulties in undertaking research with this population.

### Recruitment

We explored how people unemployed through chronic pain might be identified. Most of the clients interviewed had been referred for IPS from the job centre or existing employment programme. However, participants identified a range of other recruitment opportunities, including GP practices, chronic pain services, physiotherapy, rheumatology, support groups, community groups, and libraries. However, one ESW hypothesised that individuals recruited in different settings may differ from the clients who were currently offered IPS: “the referral would be different, it would be interesting to see those clients coming from a doctor’s surgery that are told to speak to someone, ‘cause their mentality might be different, to people that are in the process of referrals coming often from the job centre” (ESW5).

HCPs discussed recruiting eligible patients from healthcare settings. They felt it would be possible to undertake database searches, using the NHS digital Read codes. Read codes are the standard clinical terminology system used in general practice in the UK [27]. They provide a hierarchical clinical coding system for the purpose of reporting, research, decision-making, and to allow data to be shared reliably between different computer systems, and have been used previously for chronic pain [28]. However, the HCPs recognised that there is currently no Read code for unemployment that could be searched so that the searches could turn up patients with pain and certified sickness absence, but not unemployment. HCPs suggested that Read codes for chronic pain conditions could be used in conjunction with medication codes (e.g. opioid or gabapentinoids) to identify potential participants.

One client expressed concern about recruitment in healthcare settings: “I don’t think... if I got a letter like that from my GP, I think I would just shove it in the bin. Or I would.. I wouldn’t be very happy.” (C5).

HCPs also felt they could personally identify individuals who would benefit from the programme based on regular contact through primary care services. Clients felt this could be a positive way to identify people who might benefit from the intervention. “That sounds like a good idea, because in a way when I was put on my antidepressants the doctor who gave me the antidepressants and telling me what to do, he told me that I needed to be part of a support programme, you are not gonna be able to do this alone, you’ve got your church, you’ve got your wife but you are gonna need more than that. You need someone to steer you in the right direction, which I have done and I continue to do.” (C4).

There appeared to be confusion amongst clients about whether or not their enrolment in their IPS programme was voluntary or mandatory. Although the programme was explained by the ESWs as a voluntary programme, some clients reported that they did not feel they had a choice about participation particularly if they were referred by the job centre. It was evident in the interviews that the uncertainty about choice over employment services was particularly unhelpful in trying to elicit their engagement with employment-related research and whether or not they would understand that their involvement in the research was voluntary: “Even the advisors say it’s “voluntary” it’s not voluntary.. when you go to see the advisor at the job centre they said “you’re on the work activity group, you must be doing something” so they put you on [IPS programme]. And then when you come here, they say it’s all voluntary. But it’s not voluntary, the jobs centre put you on this course for a reason.” (C3).

Clients also reported that there was a lot of paperwork regarding the programme and evaluations of the IPS that were being conducted (unrelated to the current research) and that this was not clearly explained to them. One client complained about the volume and content of questions asked in evaluations, not seeming to understand the purpose: “Yeah it’s like these [consent forms and information sheets]. Tick this thing on the computer. Tick, tick, tick. One question was did you have school dinners as a child? Yeah what’s that about?” (C3).

### Intervention

Clients and ESWs were aware that there are multiple employment interventions available for unemployed people. However, IPS was felt to be the best approach for patients with chronic pain, as other employment interventions were seen as not appropriate for individuals with complex issues “the other programme, they understood my problems but they didn’t do anything about it” (C4).

The ESWs interviewed were currently working on an IPS programme that recruited individuals with a long-term health condition after 2 years of unemployment. All stakeholders suggested that the sooner someone could enter the programme, the better it would be. “I think the sooner you can get someone back to work, the better.” (HCP4). Within the interviews, it was explained that the pilot IPS trial would aim to recruit individuals after 3 months of unemployment. ESWs, HCPs and clients regarded this as beneficial. “Because everybody needs that helping hand, if you think about it, if somebody’s just come out of work, for 3 months and they’ve got nothing, that’s where they’re starting to lose the point, ‘I’m unemployed, I’m signing on, I’ve gotta do this, I’ve gotta do that… you know where do I go next?’ We don’t want that person to go 3 months after that, 6 months unemployed and still going nowhere, they need to be somewhere where they get the support, yeah I totally agree with that.” (C4). Clients and ESWs also felt the IPS should continue to be available for longer than a year as it could take longer to get someone ready for employment and into employment.

The crucial role of the ESW in successful IPS was apparent. Clients and ESWs felt that their relationship was crucial to finding clients employment. Moreover, some clients described little stability in the programme and felt they had not developed a relationship with a single ESW, having met multiple different ones, which hindering their experience of the programme. “I’ve just been moved from one advisor to another advisor, to a new one” (C2). This highlights that, for a successful trial, the relationship between clients and ESWs must be a key ingredient within the intervention, and any future RCT should ensure that continuity and relationship building is a guaranteed element.

ESWs discussed tailoring IPS to their clients. However, they seemed to be attempting this based on discussion with clients, with limited knowledge about the impact and management of specific conditions. “you should have advisers that have training around chronic pain and there should be a fully comprehensive directory of signposting people” (ESW4). It was acknowledged that personalised advice and a holistic approach was a key part of the intervention. Clients reported that ESWs asked about their potential needs and barriers to create an individualised plan. One client recalled that, although broadly her disability and illness had been discussed, the discussion had not included the specifics of her illness: “So, for people with chronic pain, or anybody with mental health, or, you know, or anything that’s specific, I think what would improve this service is having an advisor who specialised in that condition. Or in a couple of things. It’s having that understanding, it bridges that gap.” (C5). As the proposed trial would focus on clients with chronic pain, these comments suggest that it may be important to have training about chronic pain for ESWs so that the intervention can be appropriately tailored.

Reviewing IPS as an intervention, it was seen to be valuable and the right intervention to research, as compared with other employment interventions. Participants felt the intervention should be available sooner after unemployment started than is possible in their current programme. Issues surrounding ESWs knowledge on chronic pain and lack of continuity of contact with ESWs may be important to address before a full trial.

### Treatment as usual

It was explained to interviewees that the pilot trial lists were proposing to give out a booklet to those in the trial who were randomised to the “treatment as usual” group, and the proposed booklet was reviewed. Overall, the booklet was seen positively. HCPs valued the booklet answering common questions, being reader-friendly and in lay language. It was seen that too much information could be overwhelming. “I think it’s very useful, I think it will help people…I think patients will find it quite useful.” (HCP8). HCPs reported willingness to randomise patients to both the IPS and control interventions. One HCP explained that in previous studies, participants had been unhappy if they had been randomised to a control group, and felt that it would be important to explain the benefit of both interventions. “I tend to.. not sell it if it’s a non-intervention or control, but actually saying.. you are actually really important in this study as well. They are part of it and they are helping. If we get to the actually nitty gritty and we are actually recruiting patients, then they’ve got that far, and we’ve talked about it, and we’ve consented them, then hopefully they are still on board.” (HCP4).

### Outcomes

We asked interviewees for their thoughts about outcomes from the intervention that should be assessed. Participants appreciated that the outcome of the programme would be entry to employment. However, clients and ESWs also pointed out that, because of the length of the intervention, clients may have not yet found employment. ESWs were keen to talk about the success stories of the IPS programme, with clients improving in several domains of quality of life. In the current study, none of the clients interviewed had yet found paid employment, but all were keen to discuss the progress they had made. Clients and ESWs highlighted that clients gained skills from the programme that made them qualified for employment, and “job readiness” was seen as a potential outcome for a future study. “I feel a lot better about starting now, I don’t expect anything to happen until I’ve been here a few months or so, one thing at a time, I need some solid ground to stand on” (C7).

HCPs and ESWs noted it was important to build clients’ confidence. The knee jerk reaction, which throws up the barriers in the first place. So you’ve got to get those down. And by doing that, when people talk about it, when you are chatting with the client and I’ll say “but if you are doing that, why don’t you take it.. it’s the same thing but you are on a different..” and they go “ohhh right”. Erm, so you are getting those kind of barriers down. At the same time as doing that, cos you actually understand, it builds their confidence. And “maybe I could do that, maybe I could plan” (ESW3). Some clients also described a boost in their confidence since starting the programme. Participants also discussed changing clients’ attitudes and views about work. HCPs expressed concern that some patients may not want to gain employment, and it would be important to identify and measure motivation as part of the study.

HCPs postulated a number of benefits of employment for individuals with chronic pain. Employment could increase physical activity which might reduce pain levels. Additionally, they highlighted the link between chronic pain and mental health, stating that improving social interaction and sense of achievement through work could improve depression. Overall, it was thought that employment could improve patients’ overall quality of life. “So then at least these sort of work based programmes are starting to tackle that, helping people to get out of the house regularly, introducing some sort of ‘maybe I could do something’, a degree of hopefulness, where there is a degree of hopelessness” (HCP8). Clients themselves reported that they were being more active in daily life, “it gets me out of the house and that you know what I mean, well… I’m out and about every day.” (C6).

A summary of all the barriers and facilitators described by the participants is included in Table 2

**Table 2:** Barriers and facilitators to a future trial of individual placement and support recruiting in primary care as identified by the key stakeholders: clients, Employment Support Workers and primary care healthcare teams

	Barriers	Facilitators
**Doing a trial**	**Clients:** Is it voluntary to take part or compulsory?	**All:** This is a good thing to test. It is important.
**Recruitment to a trial**	**Clients**: Recruitment might be challenging in primary care as our GPs do not know that we are employed/not employed. **HCPs:** There is a risk of sending a lot of letters to people with chronic pain who currently are IN WORK. **Clients:** I would not be happy if my GP wrote to me about a job intervention	**Clients:** Lots of opportunities to find unemployed people with chronic pain: Job Centre, from other employment programmes, chronic pain services, Physiotherapists, Rheumatologists, Support groups, Community groups, Libraries **HCPs:** We can find people using Read code searches of the primary care database. Although no code for “unemployed”, we can use chronic pain and medications e.g. opioids **HCPs:** We know who these patients are personally **ESWs:** Recruiting from places other than the Job centres might bring in people who are different and perhaps better motivated **Clients:** I would be more likely to consider this if my GP recommended it for me
**Acceptability of the intervention**	**Clients:** It needs to be clear that it is a choice to go on the programme that it is not mandatory and that you are not being “forced” into work by anybody **ESWs:** Clients need to be motivated to want to work for this intervention to be possible **ESWs:** Clients sometimes need more than 12 months support to be ready to apply for competitive employment	**Clients:** A trial offering this support earlier after you have lost your job would be likely to be much better for people before they have lost confidence etc.
**Delivering the intervention**	**ESWs:** We would need to know more about chronic pain and chronic pain services and management to do this **Clients:** ESWs would need extra training in chronic pain	**Clients:** The relationship with the ESW is crucial for this and it works best when you have continuity and build a relationship
**Process**	**Clients:** There is a lot of paperwork already involved in IPS assessments	
**Acceptability of the TAU**		**All:** The booklet provided for “Treatment as Usual” is brilliant. **HCPs:** I think it would be very helpful and would be happy to recommend patients if they could have this OR the treatment
**Outcomes that are important**	**HCPs**: Motivation to work will be an important factor determining outcome	**Clients:** Although your main reason for attending is to get a job, you get so much more out of it, e.g. confidence, increased social interaction **ESWs:** The clients develop over time; they are not all “ready” for a job at the same stage but you see them benefitting in other ways to begin with. “Readiness for work” could be an important outcome. **HCPs:** The benefits will include less pain, more exercise, less depression, better quality of life, not just a job

## Discussion

This study explored the barriers to, and facilitators of, a future RCT of IPS for people unemployed with chronic pain, through the perceptions of the three key groups of stakeholders. Overall, all groups were positive about the relevance and importance of a trial in this client group and pointed to IPS as the “right” occupational rehabilitation intervention to test. A key challenge identified was recruitment in primary care particularly given the lack of employment status information in existing UK healthcare databases. Clients expressed concern that their GP would not know about their employment. HCPs identified a number of possible strategies for recruitment: Read code searches, hand searching of records, using personal knowledge, opportunistic referral and posters in waiting rooms for self-referral, each of which will be investigated in the future pilot trial. One area that became clear would require particular thought is ensuring that patients have fully understood that their participation in research is voluntary and that they can withdraw consent at any time. The interviews revealed uncertainty in the minds of current IPS clients as to whether or not their engagement in the Solent Jobs Programme was voluntary, and this appeared to lead to further confusion when the concept of research was introduced. Finally, training specific to chronic pain for ESWs working with trial participants was thought to be important as was accessibility and long-term relationships with the ESW. All stakeholders viewed a trial as feasible.

This study had a number of strengths in that it sought to obtain the views of all three key stakeholder groups, and to obtain a diverse range of views, sampling until saturation was reached. Data collection, coding and transcribing were done by two experienced researchers. However, there were a number of limitations: IPS is not yet routinely available across the UK. The study benefitted from a local programme with sufficient experience of supporting clients through IPS to take part, but this was still, of necessity, a relatively small sample from one geographical location. Clients for this study were identified and given written information about this study by ESWs. Care was taken not to explain too much about the study objectives to the ESWs in advance, but we cannot exclude the possibility that those who agreed to participate were in some way “different” from other clients, and it is likely that those who agreed were more positively disposed to research and perhaps had derived more benefit from the IPS intervention than others who were not interviewed. Unfortunately, principles of data protection did not give us an opportunity to explore which people chose not to take part. Also, the views of the HCPs were elicited from two GP practices who “volunteered” to participate. We must assume that there is the possibility that the views they expressed were generally more positive about this research than would be the case if we were able to incorporate a wider number of HCPs.

The study highlighted an important challenge for a future trial in terms of obtaining and maintaining engagement with this client group. To try to maximise participation, face-to-face and telephone interviews were offered. However, high numbers of those who had agreed to participate through each means were found to be unavailable at the appointed time/place. As highlighted by the HCPs, chronic pain patients often have complex problems including low self-efficacy, poor organisational or health literacy, unpredictable symptoms and comorbid conditions such as depression and anxiety [5]. Design of any trial will need to take account of these issues, perhaps predicting a lower level of recruitment and a poorer rate of retention than that seen in different client groups and considering specific strategies to enable ongoing participation both with the intervention and the research. However, these challenges may not be different from those reported in a trial of IPS for people with serious mental illness [29].

Our findings highlighted that trial participants may need additional support and explanation of the purpose of the research and their rights to give, or withhold, consent to participate and withdraw that consent at any time. It appears that clients referred to the Solent Jobs Programme, a pre-condition for which is that they should be choosing to try and obtain employment, were uncertain of their agency and felt some compulsion to engage because they perceived a risk of losing their welfare benefit payments. Perhaps because of cultural differences, or differences in national health and welfare systems, no such problems were reported in the recent Norwegian pilot study [14]. In developing a UK clinical trial, researchers will need to be sensitive to this complexity and make every effort to enable participants to give written informed consent to the research. These issues are relatively under-researched [30], and it may be that a study within a trial (SWAT) is indicated to explore this matter.

The relationship between clients and ESWs is viewed as critical to the success of IPS. Clients particularly valued continuity and long-term support. However, there was a suggestion from some clients that the relationship with their ESW had been discontinuous and consequently unhelpful. IPS has an agreed set of underlying fidelity principles [31] which must be adhered to in order to maximise the chances of successful outcomes. The Solent Jobs Programme has been audited against these principles and found to be showing a fair level of fidelity, but it is vital that ongoing adherence to these principles is ensured if we are to conduct an effective future RCT. The importance of the ESW relationship has been acknowledged in another qualitative study undertaken amongst psychiatric IPS patients in an RCT [29]. Another qualitative study amongst ESWs delivering IPS identified eight essential characteristics to do IPS, including empathy, persistence, passion and hardiness, as well as team orientation, professionalism, initiative and commitment to outreach in the community [32]. Taken together, it is clear that a crucial aspect of successful IPS is the quality of the relationship with the ESW and the continuity of support for the client. Thus, in developing a trial, it will be essential to ensure fidelity of IPS to these principles and maximise the opportunities for high-quality empathic support.

The opportunity for the current research came out of the existing Solent Jobs Programme which is available through local job centres for anybody unemployed ≥ 2 years with a long-term health condition. Therefore, the clients had all experienced very long-term unemployment. It is insightful and interesting that not only ESWs, but also clients, pointed to the importance of offering IPS early after unemployment and were extremely positive that the pilot study was set to recruit after 3 months’ unemployment. Re-employment rates are well-known to be much lower after prolonged unemployment, due to a complex array of factors including physical and mental health impacts, loss of confidence and self-efficacy, de-skilling and financial dependence on welfare benefits [33–38]. The views expressed reinforce our own view that a future trial needs to investigate early IPS particularly given that the Norwegian trial is recruiting pain patients with > 2 years’ unemployment [14].

All stakeholders described a range of outcomes that could be improved by IPS including mood, social activity, physical exercise, self-efficacy, confidence, readiness to work and health-related quality of life. For most published IPS trials, competitive employment has been the principal outcome [11], but the current findings, alongside some from other research, indicate the importance of considering the impact of this intervention across the range of physical and mental health domains and suggest that its effectiveness and cost-effectiveness will need to be carefully assessed. It is clear that the pilot trial will need to collect relevant information to allow assessment of the health and quality of life outcomes which are most responsive to change but are also meaningful to patients.

## Conclusions

- People with chronic pain, healthcare providers and employment support workers all see an important justification for doing trials of vocational/occupational interventions in people with chronic pain who are unemployed.
- All stakeholders thought that a trial of Individual Placement and Support (IPS) in chronic pain patients who are unemployed was potentially feasible but pointed to important considerations around methods of recruitment and assessment of motivation to obtain work.
- A key element of the success of IPS is a supportive, trusting one: one relationship between client and employment support worker. Caseloads will need to be appropriate to guarantee this.
- Stakeholders see many potential gains from the IPS intervention including achieving a healthier lifestyle, improving mental health, improving quality of life as well as employment itself.

## Data Availability

Upon completion of the feasibility programme, data will be available upon request from the corresponding author.

## Abbreviations

IPS: Individual Placement and Support
ESW: Employment Support Worker
RCT: Randomised controlled trial
HCP: Healthcare professional
COREQ: Consolidated Criteria for REporting Qualitative studies
